# Special Issue: Molecular Biomarkers in Solid Tumors

**DOI:** 10.3390/genes12070984

**Published:** 2021-06-28

**Authors:** Nicola Fusco, Caterina Marchiò, Michele Ghidini, Cristian Scatena

**Affiliations:** 1 Department of Oncology and Hemato-Oncology, University of Milan, 20141 Milan, Italy; 2 Division of Pathology, IEO, European Institute of Oncology IRCCS, 20141 Milan, Italy; 3 Department of Medical Sciences, University of Turin, 10124 Turin, Italy; 4 Division of Pathology, Candiolo Cancer Institute FPO-IRCCS, 10060 Candiolo, Italy; 5 Division of Medical Oncology, Fondazione IRCCS Ca’ Granda—Ospedale Maggiore Policlinico, 20122 Milan, Italy; 6 Division of Pathology, Department of Translational Research and New Technologies in Medicine and Surgery, University of Pisa, 56126 Pisa, Italy

Diagnostic strategies using a next-generation systematic approach have the potential to radically improve the outcome and subsequent quality of life of patients with cancer. Due to unprecedented technological developments, coupled with novel multidisciplinary approaches in translational research, a multitude of biomarkers at genomic, transcriptomic, proteomic, and immunologic levels have been discovered and validated [1]. These biomarkers are strongly impacting pathology and treatment decision-making in oncology. We edited the present Special Issue of *Genes* on “Molecular Biomarkers in Solid Tumors” to provide a snapshot of newly available and/or bona fide biomarkers for the clinical management of patients with cancer. We aimed to offer a pathology-oriented angle on some of the hot subjects regarding biomarker-based oncology. Particular attention has been paid to the selection of articles focusing on tailored biomarkers assessment in terms of technology and analytical strategies. The present collection that we had the pleasure to edit consists of three original research articles and eight reviews covering different biomarkers in different types of cancer. 

Four articles provide insights into the role of specific biomarkers in cancers using a histology-agnostic approach. Among these, original research by Gianluca Lopez and collaborators describes the prevalence and prognostic impact of somatic mutations in leucine-rich repeat kinase 2 (LRRK2), a major contributor to familial Parkinson’s disease, in different neoplastic settings [2]. Through the analysis of molecular data from 14,041 tumors available at cBioPortal.org, they report a significantly different risk of death based on LRRK2 copy-number variations (CNV). These results may pave the way for new primary and translational research studies to clarify the role of LRRK2, not only in neurodegenerative disorders, but also as a biomarker in oncology. Another interesting study by Khan and Sarkar provides an elegant overview of the oncogene astrocyte elevated gene-1/metadherin (AEG-1/MTDH) role [3]. This work has been particularly welcomed by the Editorial Board, given that since its initial cloning in 2002, many studies corroborated the role of AEG-1/MTDH in different neoplastic settings. We believe that this wide range of information is thoroughly analyzed by the authors. Similarly, many studies have recently provided evidence of the oncogenic activity of contactin 1 (CNTN1), a cell adhesion molecule, in a variety of cancer types. In this respect, Gu et al. clarify the current state of knowledge on CNTN1 biochemical mechanisms and interactions in various signaling pathways frequently altered in cancer, including the phosphatidylinositol 3-kinase (PI3K)/protein kinase B (Akt)/mammalian target of rapamycin (mTOR) [4]. Another gene that is deeply involved in this pathway is represented by the tumor suppressor phosphatase and tensin homolog (PTEN). Despite the actionability of PTEN alterations, their role as biomarkers remains controversial in clinical practice. In this Special Issue, we also present an update on the biologic and genetic alterations of PTEN across the most frequent solid tumors, as well as on their actual and/or possible clinical applications [5].

Then, we introduce a series of articles focusing on selected types of tumor. The Australian group of Allegra Freelander and colleagues summarizes the currently available data on prognostic, predictive, and pharmacodynamic molecular biomarkers in one of the most frequent types of tumor worldwide: the hormone receptor-positive breast cancer [6]. Another big killer is addressed by Ouyang et al. in an original research article taking advantage of multi-omics data analysis in liver cancer [7]. In this study, they propose an integration of biostatistics methods for biomarker identification in an unbalanced dataset of hepatocellular carcinomas. With this approach, the authors obtain 34 differentially expressed genes that may improve the pathological diagnosis of this tumor type. In the digestive system, pancreatic cancer is also a highly lethal condition due to its aggressiveness and limited treatment options. An international team of scientists summarizes the current knowledge about novel immune-based therapies in this type of tumor, using microsatellite instability (MSI) as a predictive biomarker [8]. We would like to highlight that immunotherapy has also been widely explored in glioblastoma during the past few years. This tumor consists of an admixture of malignant cells and stromal cells within a variably dense immune microenvironment. A review article that one of the Editors prepared for this Special Issue attempts to elucidate the complexity of glioblastoma microenvironment composition, highlighting the current state of the art in immunotherapy approaches [9]. Then, we selected a research article on cutaneous squamous cell carcinoma (SCC). Despite it being the second-most-common skin cancer in Caucasians, there is still a substantial lack of clinically reliable molecular biomarkers for this tumor. Hence, only clinicopathological prognostic markers are commonly used in the clinical workup of SCC, including tumor thickness and size, depth of invasion, perineural invasion, degree of differentiation, location, stage. For this reason, we have applauded the study by Campos et al. on the prognostic significance of p53 expression and RAS mutations in cutaneous SCC [10]. Immunohistochemistry for p53 and mutational profiling for RAS on a retrospective series of 162 lesions from 128 patients revealed that p53 overexpression and RAS mutations are a bona fide predictor of recurrence and local aggressiveness, respectively. Finally, we would like to offer to the readership of this Special Issue two articles on malignancies affecting the urinary tract. A review by Cinque and colleagues provides insights into the role of circulating RNA (i.e., miRNA, lncRNAs, and circRNAs) as possible diagnostic and prognostic biomarkers of renal cancer [11], while De Lorenzis et al. stress the importance of high-throughput genomic characterization in upper urinary tract urothelial carcinomas [12].

Our understanding of the molecular landscape of solid tumors is expanding day by day. We believe that a biomarker-based approach at a single-patient level in the risk assessment and treatment selection would represent a quantum leap for the community of healthcare providers and patients.
